# Crop model ideotyping for agricultural diversification

**DOI:** 10.1016/j.mex.2021.101420

**Published:** 2021-06-19

**Authors:** Eranga M. Wimalasiri, Ebrahim Jahanshiri, Vimbayi Chimonyo, Sayed N. Azam-Ali, Peter J. Gregory

**Affiliations:** a Crops For the Future UK, NIAB, 93 Lawrence Weaver Road Cambridge, England, United Kingdom; b Department of Export Agriculture, Faculty of Agricultural Sciences, Sabaragamuwa University of Sri Lanka, Sri Lanka; c Centre for Transformative Agricultural and Food Systems, School of Agricultural, Earth & Environmental Sciences, University of KwaZulu-Natal, South Africa; d School of Agriculture, Policy & Development, University of Reading, Earley Gate, Reading, United Kingdom

**Keywords:** Aquacrop, Big environmental data, Calibration, Crop modelling, Hemp, Ideotyping, Underutilised crops

## Abstract

Evidence based crop diversification requires modelling for crops that are currently neglected or underutilised. Crop model calibration is a lengthy and resource consuming effort that is typically done for a particular variety or a set of varieties of a crop. Whilst calibration data are widely available for major crops, such data are rarely available for underutilised crops due to limited funding for detailed field data collection and model calibration. Subsequently, the lack of evidence on their performance will lead to the lack of interest from the policy and regulatory communities to include these crops in the agricultural development plans. In order to motivate further research into the use of state of the art techniques in modelling for less known crops, we have developed and validated an ideotyping technique that approximates the crop modelling parameters based on already calibrated crops of different lineage. The method has been successfully tested for hemp (*Cannabis sativa* L.) based on a well-known crop model. In this paper we present the method and provide an impetus on the way forward to further develop such methods for modelling the performance of minor crops and their varieties.

• The approach works based on modelling the performance of hemp using the knowledge from an existing model that was developed for sugar cane.
• The customisation uses one of the most prominent models (AquaCrop) to approximate growth coefficients for hemp (*Cannabis sativa* L.).
• A sequential procedure was used to approximate the phenological stages in the growth model that performs well in the calibration and validation steps.

## Graphical abstract

**Figure:**
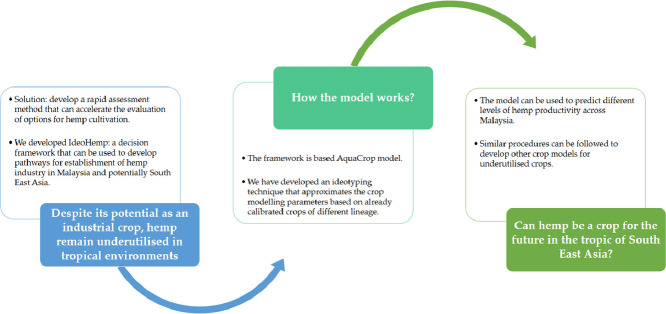

Specifications table

**Table:** 

Subject Area:	Agricultural and Biological Sciences
More specific subject area:	Crop modelling
Method name:	IdeoHemp
Name and reference of original method:	P. Steduto, T.C. Hsiao, E. Fereres, On the conservative behavior of biomass water productivity, Irrig Sci. 25 (2007) 189–207. https://doi.org/10.1007/s00271-007-0064-1 D. Raes, P. Steduto, T.C. Hsiao, E. Fereres, AquaCrop—The FAO Crop Model to Simulate Yield Response to Water: II. Main Algorithms and Software Description, Agron J. 101 (2009) 438–447. https://doi.org/10.2134/agronj2008.0140s.
Resource availability:	This crop modelling exercise was implemented using the data collected from literature which are publicly available. All the information are cited in the reference list.

## Background and study rationale

Crop models are important tools for assessing the performance of crop species or cultivars in regard to different management practices and growing conditions [1]. With the increasing concern over climate change, land use and potential utilization of unexploited crops that can become crops of the future, crop modelling approaches are gaining popularity. Currently, several crop models with different levels of complexity exist that are designed to simulate pre-determined and tested crop species and varieties/ cultivars. For example, the latest versions of DSSAT (v4.7.5) [2,3], APSIM (7.10) [1] and AquaCrop (6.1) [4] can simulate 42, 39 and 15 crops respectively. Most of these models however, simulate a few major crop types while neglecting minor and underutilised crops. Lack of detailed field experimental data (parameterization and validation) is a major reason for the slow pace of developing new crop models in crop modelling platforms. Furthermore, inclusion of a new crop into an existing model is rather difficult and needs extensive field work along with software development. Crop simulation modules are already established for major food and fibre crops such as maize, wheat, rice, soybean, potato and cotton [1,2,[4], [5], [6]]. The existing models can be customised using experimental data and/or secondary data gathered from the literature, where good or enough observed data are not available. Crop models are site and crop specific, therefore, their application ahead of the conditions they were originated from or tested in, can be seen as an inherent risk [7]. Furthermore, unavailability of accurate input data such as appropriate weather and soil characteristics, water balance and management factors will also limit the applicability of crop models [8]. However, data related to the growth and development of a certain crop can be easily obtained from the literature. Also, highly accurate environmental data are increasingly available for diffenet part of the world. This provides an opportunity to parameterize the models and provide an evidence for the performance of economically important but unexploited crop species that would otherwise be impossible to obtain.

Out of the few fibre crops, Hemp (*Cannabis sativa* L.) is a high potential multipurpose crop which is illegal to cultivate in many parts of the world, therefore, it has received less attention from crop modelling communities. However, more and more cases are being made for hemp as an economically viable crop, particularly in temperate and tropical environments. In December 2020, The UN Commission on Narcotic Drugs (CND) decided to remove *Cannabis* from the ‘most dangerous’ list of drugs. This, along with many initiatives worldwide, has given rise to further interest from research and policy communities. So far, no crop module is available for hemp in widely used crop models such as APSIM, DSSAT and Aquacrop. Whilst the development of hemp model in APSIM was initiated two decades ago [9], the model is still not available in the current version of APSIM (7.10). The ‘Simple, Easy to use, Modelling Language’ (SEMola) platform was calibrated for hemp and used in Italy [10]. However, this model needs extensive input data and specific knowledge for simulations. Therefore, in order to provide initial impetus to further development of hemp as a crop for the future, simulation of its growth and development needs quick attention.

Detailed soil and climate suitability assessment [11] has shown that hemp has a potential to be cultivated in tropical environments such as Malaysia [12]. However, due to the unavailability of user-friendly and simple crop models, understanding the dynamics of growing hemp in these environments remains difficult. Therefore, we propose a crop model customisation approach that can potentially be used in simulations of new crops or crops that are currently not available in crop models. The AquaCrop model [4,13] was used as a proxy in this crop modelling exercise.

## Aquacrop model

This model is an evolution of Doorenbos and Kassam's [14] initiative, published in FAO's Irrigation and Drainage Paper No.33. According to Greaves and Wang [15], in this model crop grows in a soil-crop-atmosphere environment which is characterized by the relatively small amount of input data. When AquaCrop performs the simulation function, four files are utilized, namely; soil file, crop file, climate file, and management file.

AquaCrop's main distinguishing features from previous approaches include (i) the ability to use a simple canopy growth and senescence equation to (ii) separate evapotranspiration (ET) into soil evaporation (Es) and crop transpiration (Tr), (iii) calculate yield (Y) as a function of biomass (B) and harvest index (HI), and (iv) to segregate the effects of water stress into four components – canopy growth, canopy senescence, stomatal closure and HI.

Another evolution relates to AquaCrop is the use of cumulative transpiration (Tr) and a normalized water productivity (WP) parameter to calculate biomass (B):

(1) $$B=WP^{*}\sum Tr$$

Water productivity is normalized by dividing the daily Tr. WP's normalization makes it more conservative and applicable to diverse locations, seasons and climates, and even different levels of management practices [16]. The equation runs on a daily time step [4,13], which brings it closer to the time scale of crop responses to water stress [17]. The model can also run using monthly or mean decade temperature, rainfall and ETo records which it approximates into daily time steps when running [13]. This leads to the model's simplicity which is coupled with the model's fewer input requirements relative to other crop models [4,18,19]. These properties make the model applicable in areas with limited data sets.

## Model calibration

Specific crop module for hemp is not available in Aquacrop. Therefore, the initial step was to select a suitable crop that matches the growth, development and yield of hemp. However, no crop is available in Aquacrop which is exactly similar to hemp. As a way forward, we aimed to identify an existing parameterized crop that is similar to the growth habit of hemp and calibrated the key parameters such as the canopy and harvest index (HI) attributes. To ensure a module's suitability, simulations were conducted using various crops (barley, maize, sorghum) to select the best matched growth habit. It was found that sugarcane module is the best option as it closely resembles hemp in terms of growth habit [20].

The sugarcane crop module was iterated by initially modifying the crop life cycle parameters such as phenology, including calendar days: from sowing to emergence, maximum rooting depth, flowering, beginig of senescence and maturity (length of crop cycle) and length of the flowering stage. Subsequently, where simulations disagreed with observations, the sugarcane module's parameters were modified in a sequential approach following the order proposed by Boote et al. [21]. The steps were: (1) rate of canopy development, (2) leaf area index, and harvest index and lastly, (3) onset, rate, and duration of harvest index built up. Parameter modifications were made based on a literature review. Data on phenology, canopy development and HI were sourced from Amaducci et al. [22], [23], [24]. Data from Tang et al. [25,26] were used for model validation. The crop parameters used to parameterize the hemp are summarized in Table 1.

**Table 1:** Preliminary input parameters for the hemp grain and fibre crop in AquaCrop model.

Parameter	Description	Default value	Grain	Fibre
T_base_	Base temperature ( °C)	9.0	1.5	1.5
T_upper_	Cut-off temperature ( °C)	32.0	40.0	40.0
CC_x_	Maximum canopy cover (%)	95	90	95
Zr _max_	Maximum rooting depth (m)	1.80	2.00	2.00
Zr _min_	Minimum rooting depth (m)	0.30	0.30	0.30
Canopy growth coefficient (CGC)	Increase in canopy cover (fraction soil cover per day)	0.12548	0.24917	0.11917
Canopy decline coefficient (CDC)	Decrease in canopy cover (in fraction per day)	0.07615	0.09615	0.09615
Calendar Days: from sowing to flowering	0	74	72	
Calendar Days: from sowing to emergence	–	10	10	
Calendar Days: from sowing to maximum rooting depth	60	60	60	
Calendar Days: from sowing to start of senescence	330	105	105	
Calendar Days: from sowing to maturity	365	140	140	
Length of the flowering stage (days)	–	17	17	
Length of Harvest Index (HI) build up	20	15	15	
Normalized water productivity (WP) g m^−2^	30	25	18	
HI (percentage)	35	23	100	
Positive effect of HI as result of limited growth in vegetative period	Moderate	Moderate	Moderate	
Positive effect of HI as result of water stress affecting leaf expansion	Moderate	Moderate	Moderate	
Water stress during flowering (p-upper)	–	0.90		
Negative effect on HI as a result of water stress inducing stomatal closure	Moderate	Strong	Strong	
Aeration stress	Sensitive	Sensitive	Sensitive	
Planting date		31st July		
Plant population (plants ha^−1^)	140 000	140 000		

The data from Averinki [20,21] was part of hemp phenological datasets collected in the years 1996–1999 and 2003–2005 from separate field trials. According to Amaducci et al. [23], all trials were carried out at Cadriano Experimental station of the University of Bologna, Italy (latitude: 44°33″ North; longitude: 11°21″ East; altitude: 32 masl). The model was developed and tested using meteorological and phenological data from medium maturing hemp cultivars of different origin, sexual type and maturity group (See Table 2 in Amaducci et al. [23]. Trials from Tang et al. [25,26] were carried out at the research facilities of the Università Cattolica del Sacro Cuore (45°00″ N, 9°10″ E, 60 masl; Piacenza, Italy). Both experimental sites were located in the same bioclimatic conditions.

**Table 2:** Calibration and validation results for observed and simulated outputs for grain and fibre hemp for final biomass and yield.

	Observed (t ha^–1^)	Simulated (t ha^–1^)	RSME (t ha^–1^)
**Calibration**			
*Grain crop*			
Seed yield-2014	1.8	2.1	0.1
Biomass-2014	7.1	8.3	1.2
*Fibre crop*			
Biomass-2014	11.4	13.3	0.2
**Validation**			
*Grain crop*			
Seed yield-2015	2.1	2.2	0.1
Biomass-2015	9.8	9.5	0.3
*Fibre crop*			
Biomass-2015	12.5	12.3	0.2

### Climate

The climate file requires input files of maximum and minimum air temperature (*.TMP), rainfall (*.PLU) and reference evapotranspiration (*.ETo). Daily weather parameters (maximum and minimum air temperature, relative humidity, solar radiation, wind speed, rainfall, and ETo) for the experiments' duration were recorded and collected from automatic weather station located within 100-m radii from the research facilities of the Università Cattolica del Sacro Cuore, Piacenza, Italy (45.0° N, 9.8° E, 60 m asl).

### Soil

AquaCrop's soil file (*.SOL) requires input parameters for soil texture, permanent wilting point (PWP), field capacity (FC), saturation (SAT) and saturated hydraulic conductivity (Ksat). Tang [27] described the soils at the Università Cattolica del Sacro Cuore's research facilities as deep clay loam soils with good drainage. The default clay loam soil file in AquaCrop was selected.

## Method validation: model performance

**Fig. 1:**
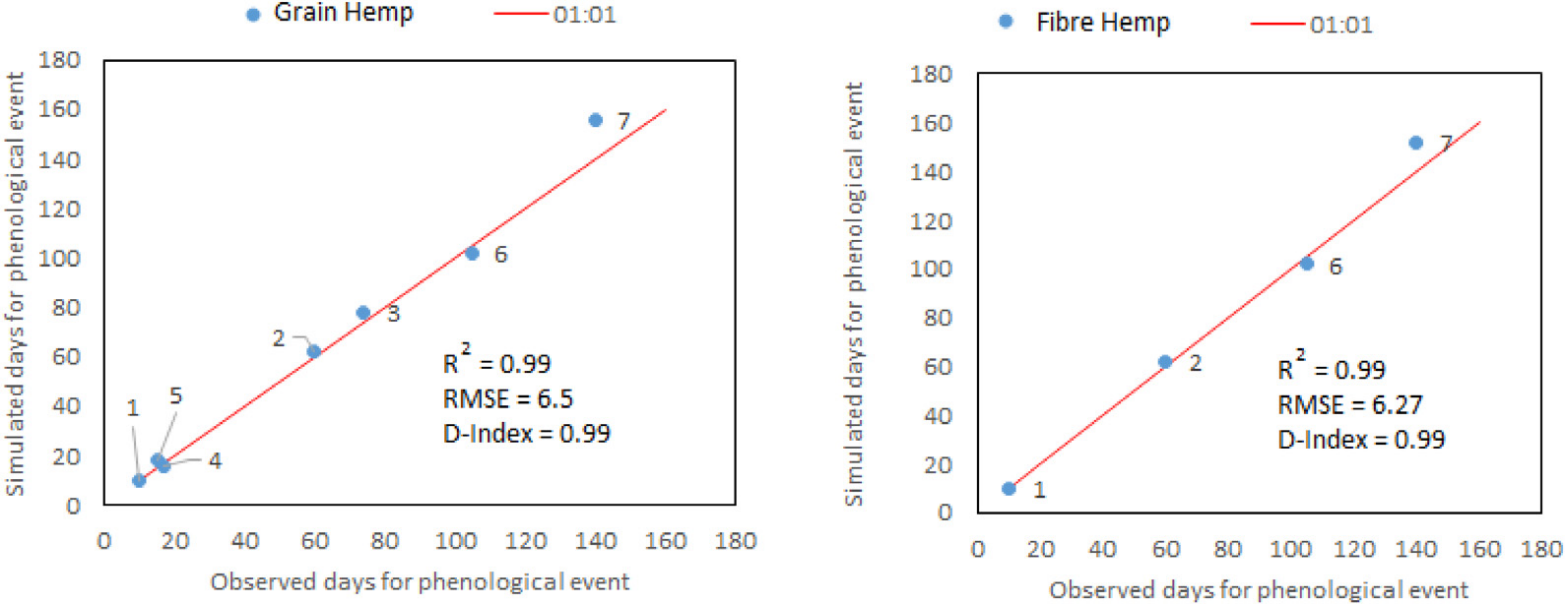
Comparison of observed and simulated values for grain and fibre hemp for phenology model validation. The numbers represent the phenological phase as described in AquaCrop. Number 1–6 and 7 represent the number of days for sowing to emergence, sowing to maximum rooting depth, sowing to flowering, length of the flowering stage, length of Harvest Index (HI) build-up, sowing to start of senescence and sowing to maturity respectively.

For model calibration and validation, crop simulation model was evaluated by comparing simulated versus observed values for grain yield and biomass. Data to validate the model was sourced from Tang et al. [25,26]. The crop models were evaluated using root mean square error (RMSE). The simulation was considered excellent when RMSE < 10%, good if 10%–20%, acceptable or fair if 20%–30%, and poor if >30% of the observed mean [28,29]. Table 2 shows the summary of the model performance. The observed and simulated values for grain and fibre hemp for validation of model phenology is shown in Fig. 1.

**Fig. 2:**
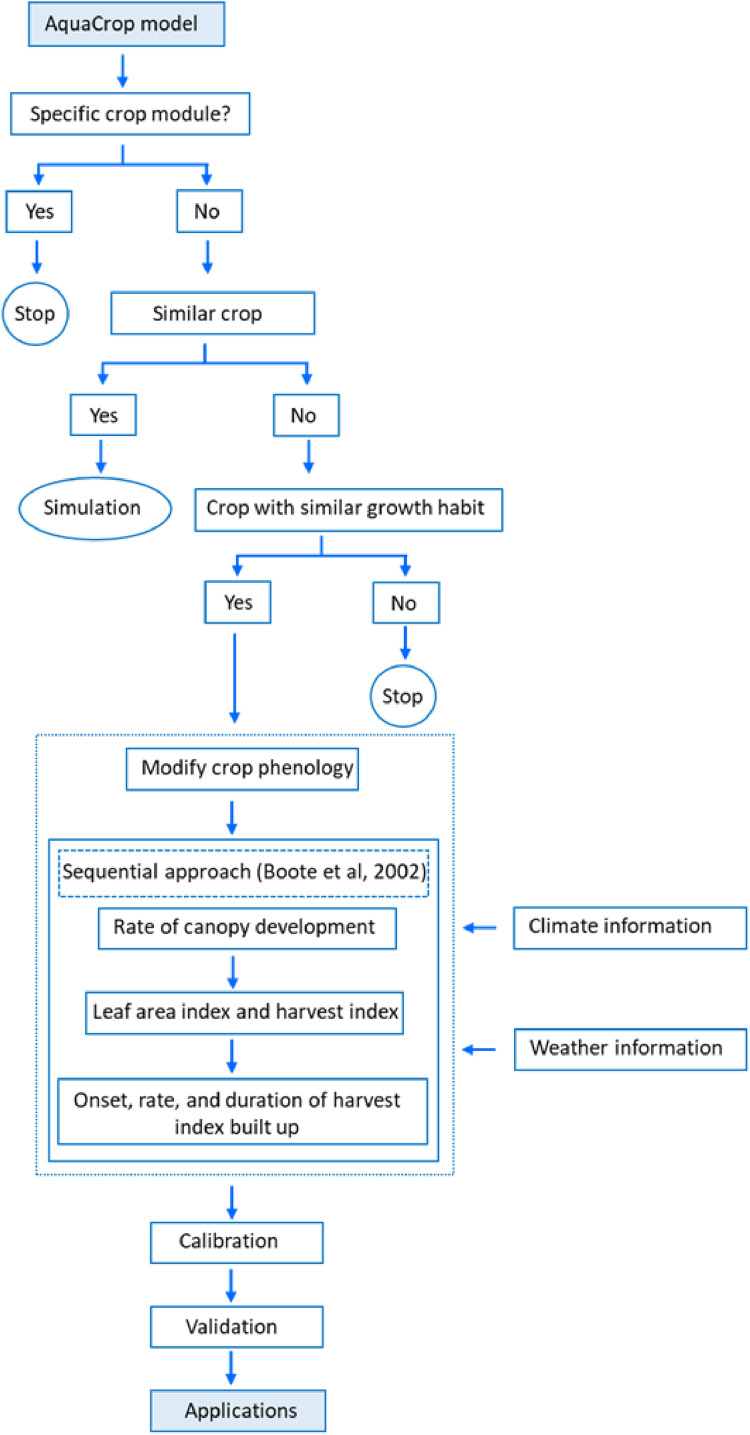
Process flow of crop model ideotyping.

The general process flow of crop model idoetyping is shown in Fig. 2. The model was successfully applied to simulate hemp yield under current and future climates and yield mapping [12]. The detailed model applications are described in Wimalasiri et al., 2020 (INDCRO-D-20–06,680) [12].

## Conclusion

Modelling the performance of a crop provides valuable initial information for the economic performance of the crop at a particular location. Modelling the underutilised crops has been difficult due to unavailability of data and robust calibration methods. This article shows basic steps that were followed to parameterise the Aquacrop model for hemp as an exemplar underutilised crop for the tropics. Following a literature review and modelling campaign, we chose a combination of crop and phenology that closely resembles that of hemp. The model was then parameterised and evaluated for both hemp seed and fiber using the data collected from the literature. The calibrated model can now be used to predict initial levels of hemp productivity across Malaysia given that environmental data such as weather and soil is available. Similar procedures can be followed to develop other models for underutilised crops in locations where no insight about the crop performance is available using data that are collected from literature.
